# The Benefits of Badminton in the Inhibition of Myopia Progression

**DOI:** 10.3390/life15050734

**Published:** 2025-05-01

**Authors:** Joanna Zawistowska, Wojciech Pawłowski, Dominik Maślach, Michalina Krzyżak, Katarzyna Rogulska, Julia Zawistowska, Aneta Narel, Alina Bakunowicz-Łazarczyk

**Affiliations:** 1 Department of Pediatric Ophthalmology and Strabismus, Medical University of Bialystok, 15-089 Bialystok, Poland; 2 Department of Public Health, Medical University of Bialystok, 15-089 Bialystok, Poland; 3 Department of Hygiene, Epidemiology and Ergonomics, Medical University of Bialystok, 15-089 Bialystok, Poland; 4 Independent Researcher, 15-256 Bialystok, Poland

**Keywords:** myopia progression, pediatric ophthalmology, axial eye elongation, vision health

## Abstract

Background: Myopia progression in children is a growing public health concern, with increasing evidence suggesting that lifestyle factors may influence its development. This study aimed to evaluate the potential benefits of regular badminton activity in slowing myopia progression in children. Methods: We analyzed data from 40 children aged 7–14 years with myopia ranging from −1.50 D to −6.25 D who participated in supervised badminton training (three 45 min sessions per week) for 10 months. A control group of 62 myopic children who did not engage in regular physical activity was included. Ophthalmological assessments were performed at baseline and after 10 months. Results: The axial length increased by 0.37 mm in the badminton group compared to 0.56 mm in the control group. The mean change in the spherical equivalent was −0.52 D in the badminton group and −0.84 D in controls. Myopia progression was slower in the group participating in regular physical activity. Conclusions: Regular badminton activity may help reduce the rate of axial elongation and myopia progression in children. These findings support the inclusion of physical activity as a complementary strategy in managing pediatric myopia. However, further research is needed to determine whether this effect is specific to badminton or reflects the general benefit of physical activity.

## 1. Introduction

Myopia is a refractive anomaly characterized by a disproportion between the optical power of the eye’s refractive components, primarily the cornea and lens, and the axial length of the eyeball. This imbalance prevents incoming light rays from converging precisely on the retinal plane, resulting in blurred vision. The predominant form, axial myopia, arises due to the excessive elongation of the eyeball, leading to progressive deterioration in visual acuity [1]. This also results in pathological changes in the growing eyeball that can lead to complications including retinal detachment, cataracts, open-angle glaucoma, and other vision-threatening diseases. These complications can lead to impaired educational and employment opportunities and reduced quality of life [2].

Globally, myopia is one of the most common causes of reduced visual acuity in children and adolescents [3]. Considering the increasing prevalence of myopia, it is crucial to understand its pathogenesis and identify potential interventions to slow its progression.

A growing number of studies describe the potential correlation between physical activity and myopia. Various sports have been examined in this context, including table tennis, tennis, basketball, football, tai chi, and rope skipping [4,5,6]. These activities differ in their intensity, environment, and visual demands, but many share dynamic features such as frequent changes in focus, rapid gaze shifts, and coordinated eye–body movements. Among them, racquet sports—particularly badminton and table tennis—have received special attention due to their emphasis on visual tracking and hand–eye coordination. Badminton, in particular, requires rapid adjustments in accommodation and convergence, along with full-body movement, which may contribute to beneficial visual stimulation.

The aim of this study was to evaluate whether regular participation in a structured badminton training program could slow the progression of myopia in school-aged children. We hypothesized that children engaged in regular badminton training would show a significantly lower increase in their axial length and a smaller shift in their spherical equivalent compared to peers who did not participate in any form of regular physical activity.

## 2. Materials and Methods

### 2.1. Study Design

This was a retrospective observational study based on the analysis of routinely collected clinical data from children diagnosed with myopia and followed at the Department of Pediatric Ophthalmology and Strabismus of the University Children Clinical Hospital, Medical University of Bialystok. All ophthalmological examinations were performed as part of routine clinical care. Group assignments were based on a retrospective analysis of medical documentation and information provided by parents or guardians during routine visits. The study was supported by a grant from the Podlaskie Voivodeship. Written informed consent for study participation was obtained from the participants or legal guardians of subjects under 16 years of age. The study was conducted in accordance with the Declaration of Helsinki Guidelines for Biomedical Research Involving Human Subjects. The study was approved by the local Ethics Committee of the Medical University of Bialystok, Poland (No. APK.002.77.2020).

### 2.2. Participants

The current study involved a total of 102 participants. The treatment group consisted of 40 children (19 girls and 21 boys) aged 7–14 years (median of 12.4 years; Q1 = 10.4, Q3 = 13.2), with refractive errors ranging from −1.50 D to −6.25 D (median spherical equivalent of −2.9 D; Q1 = −5.1, Q3 = −2.2) who voluntarily participated in a structured extracurricular badminton training program, held indoors three times per week for 45 min per session, over a period of 10 months. The control group consisted of 62 patients of the Department of Pediatric Ophthalmology (32 girls and 30 boys) of a similar age (median of 11.9 years; Q1 = 9.8, Q3 = 13.0) and refractive error (median spherical equivalent of −2.9 D; Q1 = −3.5, Q3 = −2.2) who did not engage in regular physical activity.

### 2.3. Ophthalmological Examinations

In both the treatment and control groups, both the right and left eyes were measured and examined, but only the right eye (RE) was taken into account for statistical analysis.

All patients’ visual acuity was measured when wearing their own spectacles with Snellen’s chart located at a distance of 5 m for distant visual acuity (DVA) and of 33 cm for near visual acuity (NVA). The Ishihara test was performed for the detection of red–green color deficiencies. Objective measurements of refraction were performed using an autorefractor/keratometer/tonometer Nidek Tonoref III device 20 min after the administration of a 1% cyclopentolate hydrochloride ophthalmic solution (Cykloftyal, 10 mg/mL; manufacturer: Verco, Warsaw, Poland) to the conjunctival sac of each eye. Based on these measurements, the spherical equivalent (SE) was calculated using the standard formula. This value provided a simplified representation of the refractive error, allowing for the precise quantification of myopia progression. By utilizing the SE, variations in astigmatism were accounted for, enabling a more standardized assessment of refractive changes across participants.

Since the action of cycloplegics is accompanied by mydriasis, all patients underwent fundus examination using slit lamp biomicroscopy. Also, we performed manual applanation A-scan ultrasound biometry to assess the axial length (AL) of each eye.

The instrumental base of the research consisted of the following devices:

- An autorefractor/keratometer/tonometer Nidek Tonoref III;
- A Quantel Medical Aviso ultrasound scanner with a TP-01 B.V.I 11 MHz linear probe.

### 2.4. Statistical Analysis

The Shapiro–Wilk test for normality was performed separately within each group. A normal distribution (*p* ≥ 0.05) was confirmed only for the axial length variable. Descriptive statistics were presented as the mean (SD) for normally distributed data and as the median (Q1, Q3) for non-normally distributed data. Nominal variables were expressed as frequencies and percentages.

Group comparisons were made using Welch’s *t*-test for normally distributed numeric variables and the Mann–Whitney U test for non-normally distributed variables. Pearson’s chi-squared test or Fisher’s exact test was applied for categorical variables, depending on the expected frequencies. Effect sizes were reported using Hedges’ g for Welch’s *t*-test comparisons and the rank biserial correlation for Mann–Whitney U test comparisons. Correlations between numerical variables were assessed using repeated measures correlations with 95% confidence intervals (CIs).

To evaluate the effects of the age, group, and time on the axial length and spherical equivalent, two linear mixed-effects models were constructed with the patient ID as a random effect. Model 1 assessed changes in the spherical equivalent, and Model 2 assessed changes in the axial length. Both models included interaction terms between the age, group, and time. Statistical significance was set at α = 0.05. Only right eye (RE) data were included in the analysis.

Analyses were conducted using the R statistical language (version 4.1.1; R Core Team, 2021).

## 3. Results

The characteristics of both the treatment and control groups are shown in Table 1. There were no statistically significant differences in the baseline age between the treatment and control groups (*p* = 0.556).

### 3.1. The Results of the Fitting of Model 1

The Mann–Whitney test performed showed no significant effect of gender on the spherical equivalent. Therefore, there was no basis for including a gender factor in Model 1. The correlation analysis performed showed a significant negative influence of age on the spherical equivalent, with *r* = −0.78, *df* = 100, and *p* < 0.001, 95% *CI* [−0.85, −0.70]. For this reason, an age factor was included in Model 1.

Because it can be difficult to understand the results from fitting a triple-interaction regression model, the results from fitting Model 1 were interpreted in terms of the estimated marginal means and the results of a contrast analysis. The estimated marginal means (EMMs) for the predictors of the time and group and the covariate age in the linear Model 1 are presented in Table 2. The EMMs were computed at the reference age of 12 years, which represented the average age in the study sample.

The data in the table show that the spherical equivalent value decreased over time in both groups, with a more pronounced change in the control group. The results of the simple contrast analysis with estimates of significance between and within groups for the sample mean age are shown in Table 3, with the estimated effect sizes in Table 4.

From the data in Table 3 and Table 4, it appears that subjects in the treatment group had more severe visual defects in terms of the spherical equivalence factor compared with the control group at baseline. Despite the lack of significance of the differences between the groups at baseline (*p* ≥ 0.05), the effect was estimated to be large (d = 1.90). Similarly, it appeared that there were no significant differences between the groups at time 1, but the effect size between the groups fell to a low level (d = 0.33).

The correlation analysis performed showed a significant negative influence of age on the spherical equivalent, with *r* = −0.78, *df* = 100, *p* < 0.001, and a 95% *CI* of [−0.85, −0.70]. For this reason, an age factor was included in Model 1.

In both groups, the spherical equivalence parameter continued to increase significantly over time. There was a large increase in the negative values in both groups, but in terms of the effect size, this increase was 60% greater in the control group than in the treatment group.

The results from fitting Model 1 also showed that there was no significant effect of the age factor on the spherical equivalent, with *p* = 0.577.

**Figure 1:**
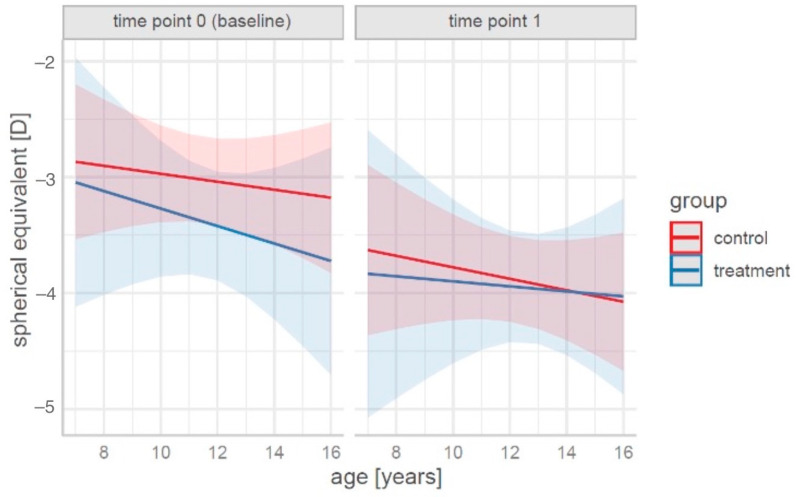
Predictions for the spherical equivalent in terms of the age, group, and time for the fitted Model 1 (solid lines represent point estimates; colored areas—*CI* of 95%).

For a graphical representation of the prediction of spherical equivalent changes according to the age, group, and time, see Figure 1.

From the data presented in Figure 1, it was concluded that systematic training slows the increase in spherical equivalent values. A particularly pronounced effect was observed in children of an older age (14–16 years).

### 3.2. The Results of the Fitting of Model 2

Similarly to in Model 1, the inclusion of additional predictors such as gender and age in Model 2 was possible provided that the univariate effects of these variables on the response variable were significant.

The Welch *t*-test performed showed no significant effect of gender on axial elongation. Therefore, there was no basis for including a gender factor in Model 2.

The correlation analysis performed showed a significant positive effect of age on axial elongation, with *r* = 0.75, *df* = 98, *p* < 0.001, and a 95% *CI* of [0.65, 0.82]. For this reason, an age factor was included in Model 2.

Similarly to in Model 1, the results from fitting Model 2 were interpreted in terms of the estimated marginal means and the results of contrast analysis. The estimated marginal means (EMMs) for the predictors of the time and group and the covariate age in the linear Model 2 are shown in Table 5. The EMMs were computed at the reference age of 12 years, which represented the average age in the study sample.

According to the estimated marginal means at the reference age of 12 years (Table 5), axial elongation increased over time in both groups, with a more pronounced change observed in the control group.

The results of the simple contrast analysis with estimates of significance between and within groups for the sample mean age are shown in Table 6, with the estimated effect sizes in Table 7.

In both groups, the biometry parameter increased significantly over time, meaning that patients’ eyes increased in axial size. There was a large increase in values in both groups, but in terms of the effect size, this increase was greater in the control group than in the treatment group.

**Figure 2:**
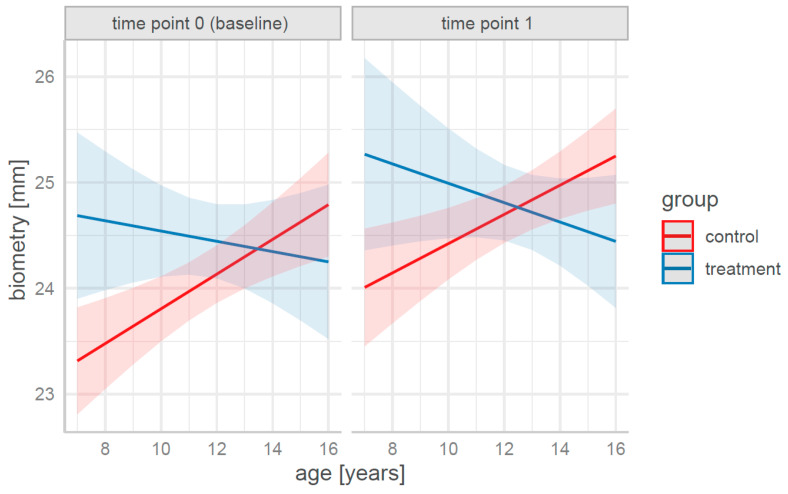
Predictions for biometry in terms of age, group, and time for fitted Model 2 (solid lines represent point estimates; colored areas—*CI* of 95%).

A graphical representation of the prediction of biometric changes by the age, group, and time can be found in Figure 2.

Although the change in biometry for children aged 7–8 years was similar between the treatment and control groups, biometry growth was 60% slower in the treatment group than in the control group at an age of 12 years and twice as slow at the ages of 14–16 years.

## 4. Discussion

Myopia must be recognized as a significant public health issue to drive necessary changes in the management of this condition, requiring a concerted effort among eye care professionals, researchers, and policymakers. With projections indicating that myopia will affect 50% of the world’s population by 2050, there is growing concern that it could become a major cause of irreversible blindness globally [3]. The increasing prevalence of myopia is further complicated by its earlier onset, which significantly raises the likelihood of developing high myopia, a condition associated with severe ocular complications, including retinal detachment, cataracts, and glaucoma. These complications pose a serious threat to vision-related quality of life, academic performance, and future employment opportunities, reinforcing the urgency of early intervention and management strategies.

In addition to its ocular complications, myopia also affects the psychological and emotional well-being of children. Our previous study demonstrated that adolescents with high myopia experience a significantly lower health-related quality of life (HRQoL), particularly in the domains of physical and psychological well-being, with girls being disproportionately affected [2]. This underscores the necessity of addressing myopia not only as an ophthalmological disorder but also as a broader public health concern that impacts overall childhood development and well-being. Furthermore, our previous research has shown that anxiety levels in children with myopia are significantly elevated, particularly among younger adolescents and boys [7]. This heightened anxiety may lead to social withdrawal, decreased participation in physical activities, and lower self-esteem, further amplifying the negative impact of myopia on children’s mental health and overall daily functioning.

Given the rising prevalence and multifaceted impacts of myopia, interest in effective interventions to slow its progression has grown substantially. Various treatments, including topical atropine and specialized spectacle and contact lenses, have demonstrated clinical efficacy in reducing myopia progression [8,9]. Additionally, lifestyle modifications have emerged as promising preventive strategies [10,11,12]. Our current research built upon this foundation by investigating the potential benefits of badminton in mitigating myopia progression. In our study, the badminton group showed a smaller mean shift in the spherical equivalent (−0.52 D) compared to the control group (−0.84 D). Axial elongation was also slower in the treatment group (0.37 mm vs. 0.56 mm). By engaging in rapid visual tracking and full-body movements, badminton may help regulate axial eye growth and reduce the risk of high myopia development. A holistic approach, incorporating both medical and lifestyle-based interventions, is crucial for addressing the myopia epidemic and minimizing its long-term impact on public health.

There are limited data on the effect of systematic physical activity on inhibiting myopia progression as the only intervention undertaken. Research suggests that insufficient time spent outdoors in childhood is one of the main modifiable risk factors for the development and progression of the defect [13]. Public health interventions that increase the amount of time spent outdoors may be the most effective prevention strategies [10,11,14,15]. There are various theories as to whether the beneficial effects of time spent outdoors are due to exposure to bright light [11,16], increased exposure to short-wavelength light (360–400 nm) [17], ultraviolet light [18], or yet other mechanisms. There are also insufficient data as to which of these is most beneficial. This gap in the research to date may make it difficult to optimize possible interventions for myopia prevention.

However, most of the time children spend outdoors consists of moderate-intensity physical activity, and studies on the impact of time spent outdoors have typically not considered this factor. It is worth noting that the training sessions in our study were held indoors, a setting that has been explored in only a few studies investigating the relationship between physical activity and myopia. These have suggested a protective effect of physical activity on the development and progression of this visual defect [5,12,19,20,21,22].

A recent network meta-analysis conducted by Liu et al. [6] evaluated the impact of various physical exercise interventions on vision health in children and adolescents. The study analyzed 17 randomized controlled trials comprising 1840 participants aged 7 to 18 years. The findings highlighted that badminton and table tennis were the most effective interventions in enhancing vision health. The beneficial effects of badminton and table tennis on vision health can be attributed to the dynamic visual demands and rapid eye movements required during gameplay. These sports involve the continuous tracking of a fast-moving shuttlecock or ball, requiring frequent shifts between near and far vision. This process actively engages the ciliary muscles, which control the eye’s focusing mechanism, thereby reducing accommodative strain and potentially slowing axial elongation, a key factor in myopia progression [23]. Although our study did not directly assess the physiological mechanisms underlying the effects of physical activity on myopia progression, it is worth noting that badminton involves the continuous visual tracking of a fast-moving object, rapid changes in gaze direction, and frequent head and body movements. These elements may provide unique visual and neuromuscular stimulation, potentially influencing ocular growth. While the potential physiological mechanisms remain speculative, the dynamic nature of badminton—characterized by frequent changes in gaze direction and coordinated body movements—may have contributed to the slower axial elongation observed in our study among children who engaged in regular training. Additionally, the increased exposure to bright lighting conditions during play might have enhanced dopamine release in the retina, which has been linked to the regulation of eye growth [24]. The combination of physical activity and enhanced visual engagement positions these sports as effective strategies for mitigating myopia progression in children and adolescents.

Several limitations of this study should be acknowledged. First, the 10-month observation period was relatively short and may not have been sufficient to fully capture the long-term course of myopia or the sustained influence of physical activity on refractive development. Extending the follow-up in future studies could allow for a more complete assessment of myopia progression patterns.

Second, the study design was retrospective and observational, with data drawn from a single clinical center. Although the two groups were similar at baseline, the lack of random assignment could have introduced selection bias and limits the ability to infer causality. Additionally, the classification into groups was based in part on information reported by parents or guardians, which may not always be fully accurate.

Third, the control group included only children who were not engaged in any regular physical activity. This makes it difficult to determine whether the observed differences were specific to badminton or related more broadly to being physically active. Comparative studies involving different types of physical activity would help clarify this issue.

Lastly, only data from the right eye were analyzed to avoid inter-eye correlations. While this is a common approach in ophthalmologic research, it may have led to the omission of useful data related to potential differences between eyes.

Although trends favoring the treatment group were observed, the lack of statistically significant differences at baseline limits the strength of causal interpretations. It should be noted that both the treatment and control groups were comparable at the start of the study in terms of their ages, refractive defects, axial lengths, and other ophthalmic parameters. Therefore, it is unlikely that the observed trends were due to initial differences between the groups. Rather, the lack of a statistically significant discrepancy may reflect the relatively short follow-up period, the limited sample size, or the natural variability of myopia progression in pediatric populations. These factors should be considered when interpreting the results and designing future studies.

Future research should aim to address these limitations through prospective, multicenter designs, the inclusion of randomized groups, longer observation periods, and broader comparative frameworks.

## 5. Conclusions

Badminton is a safe type of physical exercise that can be recommended to children with myopia. This study showed that children who participated in regular badminton training over a 10-month period exhibited a significantly slower progression of myopia, as indicated by both their spherical equivalent shift and axial elongation, compared to children who did not engage in physical activity. The observed differences were most pronounced among older participants, with axial growth at the ages of 14–16 being nearly twice as slow in the treatment group as in controls. In contrast, among the youngest subgroup (7–8 years), the axial length increased at a comparable rate in both groups.

These results suggest that regular physical training may serve as a supportive, non-pharmacological strategy in managing myopia progression in children. However, given that the control group did not participate in any form of regular exercise, it remains unclear whether the effects observed are specific to badminton or reflect a broader impact of physical activity on refractive development.

Further research, including randomized controlled trials with active comparison groups and longer follow-up periods, is needed to clarify the role of different types of physical activity and lifestyle factors in myopia control.

## Figures and Tables

**Table 1:** Characteristics of the sample at baseline by group.

Parameter	N	Group		*p* ^3^
		Control, n = 62	Treatment, n = 40	
Gender	102			0.432
Female		32.0 (51.6%) ^1^	19 (43.6%) ^1^	
Male		30.0 (48.4%) ^1^	21(56.4%) ^1^	
Age [yrs.]	102	11.9 (9.8, 13.0) ^2^	12.4 (10.4, 13.2) ^2^	0.556
Distance visual acuity [0–1]	102	0.9 (0.8, 1.0) ^2^	0.9 (0.7, 1.0) ^2^	0.485
Near visual acuity [0–1]	102	0.5 (0.5, 0.5) ^2^	0.5 (0.5, 0.5) ^2^	1.000
Color vision	102			0.020
Yes		62.0 (100%) ^1^	36.0 (89.7%) ^1^	
No		0	4 (10.3%) ^1^	
Spherical equivalent [D]	102	−2.9 (−3.5, −2.2) ^2^	−2.9 (−5.1, −2.2) ^2^	0.553
Axial length [mm]	99 ^4^	24.4 (1.3) ^5^	24.6 (0.9) ^5^	0.170

^1^ n (%); ^2^ Mdn (Q1, Q3); ^3^ Pearson’s chi-squared test, Mann–Whitney U test, Fisher’s exact test, Welch’s *t*-test; ^4^ n_control_ = 99 for axial length due to missing data; ^5^ M (SD).

**Table 2:** Estimated marginal means for factors of time and group conditioned by age (12 years) for fitted Model 1.

Time	Group	Estimated Marginal Means of Spherical Equivalent [D]	*df*	*SE*	95% *CI*
Baseline	Control	−3.04	101.1	0.19	[−3.41, −2.67]
Time point 1		−3.88	99.6	0.19	[−4.25, −3.51]
Baseline	Treatment	−3.42	99.3	0.24	[−3.89, −2.95]
Time point 1		−3.94	100.1	0.24	[−4.43, −3.46]

**Table 3:** Results of the contrast analysis for the fitted Model 1. ‘Baseline control’ refers to the reference group at time 0. All contrast effects are relative to this group unless otherwise noted.

Contrast	Estimate	*SE*	*df*	95% *CI*	*p*
(Baseline Control–Time 1 Control)	0.84	0.05	146.6	[0.73, 0.94]	<0.001
(Baseline Control–Baseline Treatment)	0.38	0.30	100.0	[−0.22, 0.98]	0.211
(Time 1 Control–Time 1 Treatment)	0.07	0.31	99.9	[−0.54, 0.68]	0.832
(Baseline Treatment–Time 1 Treatment)	0.52	0.10	162.6	[0.33, 0.71]	<0.001

**Table 4:** Estimated marginal means for the factors of the time and group conditioned by age (12 years) for the fitted Model 1. ‘Baseline control’ refers to the reference group at time 0. All contrast effects are relative to this group unless otherwise noted.

Contrast	*d*	*SE*	*df*	95% *CI*
(Baseline Control–Time 1 Control)	4.15	0.33	146.6	[3.50, 4.81]
(Baseline Control–Baseline Treatment)	1.90	1.51	100.0	[−1.10, 4.89]
(Time 1 Control–Time 1 Treatment)	0.33	1.53	99.9	[−2.70, 3.35]
(Baseline Treatment–Time 1 Treatment)	2.58	0.50	162.6	[1.59, 3.58]

**Table 5:** Estimated marginal means for factors of time and group conditioned by age (12 years) for fitted Model 2.

Time	Group	Estimated Marginal Means of Biometry [mm]	*df*	*SE*	95% *CI*
Baseline	Control	24.1	99.2	0.14	[23.9, 24.4]
Time point 1		24.7	98.0	0.14	[24.4, 25.0]
Baseline	Treatment	24.4	97.6	0.18	[24.1, 24.8]
Time point 1		24.8	98.1	0.18	[24.5, 25.2]

**Table 6:** Results of the contrast analysis for the fitted Model 2. ‘Baseline control’ refers to the reference group at the baseline time point; ‘baseline treatment’ refers to the treatment group at baseline.

Contrast	Estimate	*SE*	*df*	95% *CI*	*p*
(Baseline Control–Time 1 Control)	−0.56	0.04	146.9	[−0.64, −0.49]	<0.001
(Baseline Control–Baseline Treatment)	−0.32	0.22	98.2	[−0.76, 0.13]	0.163
(Time 1 Control–Time 1 Treatment)	−0.12	0.23	98.1	[−0.57, 0.33]	0.609
(Baseline Treatment–Time 1 Treatment)	−0.37	0.07	166.1	[−0.51, −0.22]	<0.001

**Table 7:** Estimated effect sizes for the contrast analysis for the fitted Model 2, with *σ* = 0.16. Contrast comparisons are relative to the baseline control unless otherwise specified. Negative values in this table represent the model-estimated effects where the control group served as the reference. A negative sign indicates slower progression in the treatment group relative to the controls.

Contrast	*d*	*SE*	*df*	95% *CI*
(Baseline Control–Time 1 Control)	−3.49	0.31	146.9	[−4.10, −2.89]
(Baseline Control–Baseline Treatment)	−1.95	1.39	98.2	[−4.71, 0.81]
(Time 1 Control–Time 1 Treatment)	−0.72	1.41	98.1	[−3.51, 2.07]
(Baseline Treatment–Time 1 Treatment)	−2.27	0.47	166.1	[−3.19, −1.34]

## Data Availability

Data available on request due to ethical reasons.

## Abbreviations

The following abbreviations are used in this manuscript:

DVA	Distant visual acuity
NVA	Near visual acuity
AL	Axial length
SE	Spherical equivalent
